# Docosahexaenoic Acid Decreases Pro-Inflammatory Mediators in an *In Vitro* Murine Adipocyte Macrophage Co-Culture Model

**DOI:** 10.1371/journal.pone.0085037

**Published:** 2014-01-20

**Authors:** Anna A. De Boer, Jennifer M. Monk, Lindsay E. Robinson

**Affiliations:** Department of Human Health and Nutritional Sciences, University of Guelph, Guelph, Canada; University Medical Center Freiburg, Germany

## Abstract

Paracrine interactions between adipocytes and macrophages contribute to chronic inflammation in obese adipose tissue. Dietary strategies to mitigate such inflammation include long-chain polyunsaturated fatty acids, docosahexaenoic (DHA) and eicosapentaenoic (EPA) acids, which act through PPARγ-dependent and independent pathways. We utilized an *in vitro* co-culture model designed to mimic the ratio of macrophages:adipocytes in obese adipose tissue, whereby murine 3T3-L1 adipocytes were cultured with RAW 264.7 macrophages in direct contact, or separated by a trans-well membrane (contact-independent mechanism), with 125 µM of albumin-complexed DHA, EPA, palmitic acid (PA), or albumin alone (control). Thus, we studied the effect of physical cell contact versus the presence of soluble factors, with or without a PPARγ antagonist (T0070907) in order to elucidate putative mechanisms. After 12 hr, DHA was the most anti-inflammatory, decreasing MCP1 and IL-6 secretion in the contact system (−57%, −63%, respectively, p≤0.05) with similar effects in the trans-well system. The trans-well system allowed for isolation of cell types for inflammatory mediator analysis. DHA decreased mRNA expression (p<0.05) of *Mcp1* (−7.1 fold) and increased expression of the negative regulator, *Mcp1-IP* (+1.5 fold). In macrophages, DHA decreased mRNA expression of pro-inflammatory M1 polarization markers (p≤0.05), *Nos2* (iNOS; −7 fold), *Tnfα* (−4.2 fold) and *Nfκb* (−2.3 fold), while increasing anti-inflammatory *Tgfβ1* (+1.7 fold). Interestingly, the PPARγ antagonist co-administered with DHA or EPA in co-culture reduced (p≤0.05) adiponectin cellular protein, without modulating other cytokines (protein or mRNA). Overall, our findings suggest that DHA may lessen the degree of MCP1 and IL-6 secreted from adipocytes, and may reduce the degree of M1 polarization of macrophages recruited to adipose tissue, thereby decreasing the intensity of pro-inflammatory cross-talk between adipocytes and macrophages in obese adipose tissue.

## Introduction

Adipose tissue is an active endocrine organ that secretes many proteins collectively called adipokines, which play a role in obesity-associated pathologies, such as insulin resistance and type 2 diabetes [1]. Various cells within adipose tissue, including adipocytes, macrophages, endothelial cells, and other immune cells within the stromal vascular fraction, contribute to the adipokine milieu to varying degrees [2]. Adipokines include the adipocyte-derived hormones adiponectin and leptin, as well as cytokines, such as IL-6, TNFα, IL-10 and MCP1 (CCL2) that are secreted from multiple cellular sources [1], [2]. The chronic inflammatory state in obesity is partly attributable to increased macrophage infiltration into adipose tissue, followed by increased production of pro-inflammatory cytokines, such as TNFα, IL-6, and MCP1, as well as decreased secretion of adiponectin, an insulin-sensitizing adipokine [3]. Paracrine interactions or cross-talk between adipocytes and macrophages in obese adipose tissue play a key role in the generation of the adipokine profile and can be influenced by dietary factors, such as fatty acids [3], [4].

Interestingly, saturated fatty acids are known to exert pro-inflammatory effects [4], [5]. More specifically, saturated free fatty acids like lauric acid (12:0) [6] and palmitic acid (PA, 16:0) [4] released from dysregulated adipocytes can activate toll-like receptor (TLR)-2 and TLR4 signalling respectively, which ultimately triggers NFκB-mediated pro-inflammatory gene expression and subsequent cytokine secretion from macrophages. Although negative feedback factors like suppressor of cytokine signalling 3 (SOCS3) [7] and monocyte chemoattractant 1-induced protein (MCP1-IP) [8] act to suppress pro-inflammatory cytokine signalling, these feedback factors may be dysfunctional in obese humans with type 2 diabetes [9]. In turn, macrophages activated through TLR2 [6] or TLR4 [4] signalling have been shown to undergo polarization to a unique M1-like phenotype characterized by increased lipid content and secretion of pro-inflammatory cytokines, TNFα and IL-6 [10]. These cytokines subsequently feedback onto adipocytes through paracrine signalling to sustain adipocyte-derived pro-inflammatory adipokine secretion and lipolysis [3]. This in turn sustains the increased release of saturated free fatty acids and continued TLR-signalling in macrophages [4]. Thus, in this cross-talk paradigm, dysfunctional adipocytes can be viewed as effectors secreting distress signals such as free fatty acids and chemokines, and macrophages can be viewed as the responders to these signals, which promotes their activation to the pro-inflammatory M1-like phenotype [3], [4] that characterizes obese adipose tissue [10], [11]. Moreover, the pro-inflammatory adipokine profile, generated in part through adipocyte macrophage cross-talk, is associated with decreased insulin sensitivity locally, i.e. within adipocytes [12], and peripherally, in other metabolically active tissues such as skeletal muscle and liver [13]. Thus, targeting paracrine interactions between adipocytes and macrophages as a mechanism to mitigate chronic inflammation in obesity can potentially be regarded as a therapeutic strategy.

In contrast to the effects of saturated fatty acids, long chain n-3 polyunsaturated fatty acids (PUFA), namely docosahexaenoic acid (22:6n-3, DHA) and eicosapentaenoic acid (20:5 n-3, EPA), exert known anti-inflammatory effects (reviewed by [14], [15]). Thus, increased consumption of n-3 PUFA may represent a promising strategy to reduce the production of pro-inflammatory adipokines associated with obesity. Interestingly, mice fed an obesogenic high fat diet supplemented with EPA and DHA exhibited an improved adipokine profile characterized by elevated plasma adiponectin and decreased free fatty acid levels [16], as well as decreased macrophage infiltration into adipose tissue [17]. Recently, we have shown that EPA and DHA increase adiponectin secretion and cellular protein *in vitro*, in part through activation of the nuclear receptor, peroxisome proliferator-activated receptor gamma (PPARγ) [18], [19]. Adiponectin has been shown to drive macrophage polarization towards the anti-inflammatory M2 phenotype *in vitro* and *ex vivo* [20], [21], thereby potentially re-directing the cyclic pro-inflammatory cross-talk between adipocytes and macrophages. This suggests that n-3 PUFA may beneficially modulate the obesity-associated paracrine interactions between adipocytes and macrophages. Thus, the objectives of this study were to determine if 1) DHA and/or EPA decrease pro-inflammatory adipokine synthesis and secretion and 2) the effects of DHA and/or EPA are exerted through a PPARγ-dependent mechanism. To accomplish this, we used two *in vitro* co-culture models that cultured macrophages and adipocytes either in direct contact (contact-dependent mechanism), or separated by a trans-well membrane (contact-independent mechanism), to mimic the ratio of macrophages:adipocytes reported in obese adipose tissue [22]. Here we showed that this model can be used to study the modulation of adipokine synthesis and secretion in response to various fatty acids, with or without the presence of a PPARγ antagonist.

## Materials and Methods

### Cell culture and differentiation

3T3-L1 pre-adipocytes (ATCC® CL-173™, USA) and RAW 264.7 macrophages (ATCC® TIB-71™, USA) were grown and passaged according to the manufacturer's instructions. Both cell types were maintained separately in basic media containing DMEM without sodium pyruvate (HyClone, USA), plus 10% v/v fetal bovine serum (FBS, low-endotoxin, Canadian origin, Sigma, USA), and 1% v/v penicillin streptomycin (HyClone, USA). Pre-adipocytes were seeded in 6-well plates (Corning, USA) at a density of 3000 cells/cm^2^, and at 2 days post-confluence (designated as day 0), differentiation was induced using basic media plus 1 µmol/L dexamethasone, 0.5 mmol/L isobutyl-methylxanthine, and 5 µg/mL insulin (Sigma, USA) for 2 days (day 2) as described previously [23]. Media was replaced with basic media containing 5 µg/mL insulin on days 2, 4, and 6 post-differentiation. On day 8, both cell types were serum starved with basic media containing 0% FBS for 12 hr to ensure quiescence prior to co-culture experiments on day 9. After serum starving, macrophages were co-cultured with adipocytes in direct cell contact or using a trans-well system on day 9 (described below).

### Co-culture of adipocytes and macrophages

Co-culture of adipocytes and macrophages was performed using two different methods; a direct cell contact and trans-well (contact-independent) system, with modification of methods used by Suganami et al. [3]. To set-up the co-culture on day 9, three wells of mature adipocytes were counted using a hemocytometer and trypan blue exclusion, and then averaged to get an average adipocyte count. Following this, 3.5 mL of fresh basic media containing the various fatty acid treatments were added to the adipocyte cultures. For the trans-well experiments, 2.0 mL of media was placed on the adipocytes, a 0.4 µM polyester membrane trans-well was added to the well (Corning, USA), and then 1.5 mL of treatment media was added on top of the trans-well. Next, T-75 flasks (Sarstedt, Germany) containing macrophages at 80% confluence were counted using a hemocytometer and trypan blue exclusion, spun down at 335×g at 24°C for 5 min, and then re-suspended in 2.0 mL of fresh basic media. Using the average adipocyte count, macrophages were added either directly on top of adipocytes (contact system), or indirectly onto the trans-well insert (trans-well system, contact-independent) at a dose of 17.1% of total cells; this dose represents the degree of macrophage infiltration as reported in the epididymal adipose tissue of *db/db* mice [22] and was confirmed to be pro-inflammatory relative to a lean dose of macrophages (3.8% of total cells; [22]) in pilot work (data not shown). Finally, the cells were co-cultured for 12 hr since we observed that RAW 264.7 macrophages divide after this period in co-culture, thereby offsetting the ratio of adipocytes to macrophages we aimed to study.

### Fatty acids and PPARγ antagonist treatments

For all co-culture experiments, fatty acid stock solutions of DHA, EPA and PA (≥98% pure, Cayman Chemicals, USA) were made using ethanol as a vehicle and stored at -20°C purged with inert gas. The stock solutions were freshly complexed to bovine serum albumin (BSA, ≤0.1 ng/mg endotoxin, ≤0.02% fatty acids, Sigma, USA) at 37°C prior to each experiment, and then added to basic media to a final concentration of 125 µM fatty acid:25 µM BSA. Controls received an equal volume of ethanol vehicle. The fatty acid dose was chosen based on previous work [18] that showed 125 µM of DHA or EPA maximally increased adiponectin secretion from 3T3-L1 adipocytes. Macrophage viability (assessed by trypan blue exclusion) did not differ between fatty acid treatment and co-culture conditions and exceeded 85% viability after a 12 hr co-culture period (data not shown). BSA plus macrophages co-cultured with adipocytes acted as a positive control, while BSA with adipocytes alone (no macrophages) served as a negative control. For experiments with the PPARγ antagonist, T0070907 (Cayman Chemicals, USA), the antagonist was dissolved in dimethyl formamide according to the manufacturer's instructions, and then added to fatty acid treated media to achieve a final concentration of 1 µM (IC_50_ = 1 µM with 1 nM Rosiglitazone; [24]) in the culture well. For treatments without the antagonist, an equal volume of dimethyl formamide vehicle was added to the treatment media. Each treatment condition was run in triplicate, and the experiment was independently conducted 2 or 3 times (depending on the outcome measured, see figure legends), for a final sample size of n = 6–9.

### Secreted and cellular cytokine analyses

Media was collected at 0 and 12 hr for analysis of secreted cytokine protein concentrations. At 12 hr, cells from the trans-well system (where adipocytes and macrophages could be isolated separately) were washed with 1x PBS (Sigma, USA), lysed using an All-prep Kit (Qiagen, Canada), and processed according to the manufacturer's instructions. Extra protease and phosphatase inhibitors (Roche, Germany) were added to the lysis buffer prior to use at the recommended concentrations. Total cellular protein was quantified using the bichintronic assay (Fisher Scientific, Canada), so that cellular protein could be normalized to total protein. Secreted and cellular IL-6, MCP1, IL-10, and TNFα were analyzed by Luminex xMAP technology (Bioplex-200 system; Mouse Cytokine/Chemokine Bio-plex kit, Bio-Rad Laboratories, USA). Secreted and cellular adiponectin were measured by ELISA (Quantikine Mouse Adiponectin/Acrp 30 ELISA, R & D Systems, USA) according to the manufacturer's instructions. Cellular cytokine concentrations in picograms per milliliter (pg/mL) were normalized by dividing by the lysate total protein concentration in milligrams per milliliter (mg/mL), yielding a final concentration in picograms of analyte per milligram of adipocyte protein (e.g. pg MCP1/mg total protein).

### NFκB activity assessment

Since cellular protein could be isolated from each cell type in the trans-well system, cellular protein lysates (methods described above) from adipocytes co-cultured in the trans-well system for 12 hr were used to measure NFκB activity assessed by ELISA measuring the ratio of phosphorylated-p65 NFκB (Ser 536) to total p65 NFκB as per the manufacturer's instructions (eBioscience an Affymetrix company, USA). An equal amount of protein was added to each well to normalize cellular protein between samples.

### RNA isolation and quantitative PCR

At 12 hr, cells were washed with 1× PBS (Sigma, USA), lysed using a RNeasy kit (Qiagen, Canada) and processed according to the manufacturer's instructions. Only adipocytes and macrophages from the trans-well system were lysed because cells could be isolated separately in this system. cDNA was made from 1 µg of extracted RNA using a high capacity cDNA reverse transcription kit as per the manufacturer's instructions (Applied Biosystems, USA). Real-time PCR analysis was performed using a 7900HT Fast Real Time PCR system (Applied Biosystems, USA) using the default protocol:2 min at 50°C, 10 min at 95°C, 15 s at 95°C, 60°C for 1 min, 15 s at 95°C and 15 s at 60°C for a total of 40 cycles. Primers were designed using the Universal Probe Library Assay Design Center (Roche Applied Sciences, Germany, **Table 1**) and validated primer efficiencies were between 90–105%. Samples were run in triplicate in 96-well plates, and each 20 µL reaction contained 5 µL cDNA (50 ng), 0.4 µL of 10 µM primer solution, 10 µL Power Sybr green 2× master mix (Applied Biosystems, USA), and 4.6 µL of RNase free water. All results were normalized to *Rplp0* mRNA expression, and the relative differences in gene expression between treatment groups and the pre-treatment (0 hr) were determined using the ΔΔCt method.

**Table 1:** Primer Sequences.

Gene	Forward Primer (5′-3′)	Reverse Primer (5′-3′)
*Rplp0*	actggtctaggacccgagaag	tcccaccttgtctccagtct
*Tnfα*	catcttctcaaaattcgagtgacaa	tgggagtagacaaggtacaaccc
*Il-6*	aacgatgatgcacttgcaga	gagcattggaaattggggta
*Il-10*	ggttgccaagccttatcgga	acctgctccactgccttgct
*Apn*	tgagacaggagatgttggaatg	ctttcctgccaggggttc
*Tlr4*	agaaaatgccaggatgatgc	ctgatccatgcattggtaggt
*Nfkb*	gagaccggcaactcaagac	ctcaggtccatctccttgggt
*Arg1*	gctggtctgctggaaaaactt	ccgtgggttcttcacaattt
*Mcp1*	gcctgctgttcacacagttgc	caggtgagtggggcgtta
*Nos2*	tcctgttgtttctatttcctttgtt	catcaaccagtattatggctcct
*Tgfβ1*	tcagacattcgggaagcagt	acgccaggaattgttgctat
*Mrc2* (CD206)	ccacagcattgaggagtttg	acagctcatcatttggctca
*Mcp1-IP* (Zc3h12a)	ccccaagccttccactcta	ccttgttcccatggctca
*Socs3*	atttcgcttcgggactagc	aacttgctgtgggtgaccat

### Statistical analysis

All data are expressed as mean ± SEM. The predetermined upper limit of probability for statistical significance throughout this investigation was p≤0.05, and analyses were conducted using the SAS system (SAS Institute, USA) for Windows (version 9.2). Data were subjected to one-way ANOVA (fatty acid treatment main effect) followed, if justified, by testing using Tukey's post-hoc test. For normally distributed data, Grubb's test was used to detect and remove any outliers. Data sets not exhibiting a normal distribution, as assessed by the Shapiro-Wilk test for normality, were subjected to the Kruskal-Wallis test (χ^2^ approximation) followed, if justified, by the statistical probability outcome (p≤0.05) using Wilcoxon two-sample testing. When two treatment factors were present, i.e. fatty acid treatment with either trans-well or contact co-culture effects on secreted cytokines and fatty acid treatment with or without the effect of the PPARγ antagonist on adiponectin cellular protein, data were analyzed by two-way ANOVA and followed, if justified, by testing using Tukey's post-hoc test.

## Results

### Secreted adipokine profile in the contact versus trans-well co-culture systems

**Figure 1:**
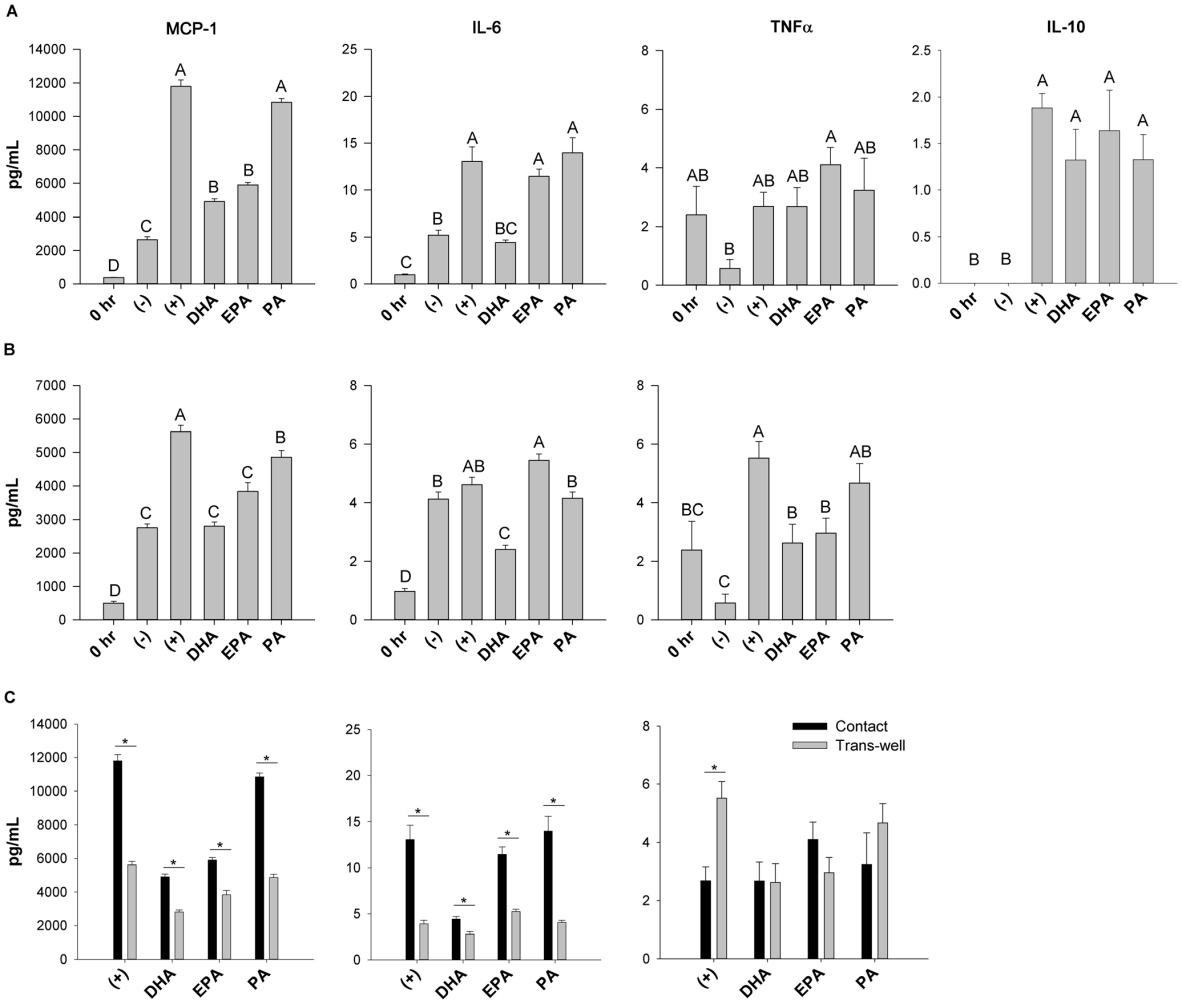
Adipokine secretion in the trans-well versus contact co-culture system. Adipokine secretion after the 12-culture incubation in the contact (A) and trans-well (B) treated co-culture conditions, and (C) a comparison of secreted MCP1, IL-6 and TNFα between the contact and trans-well co-culture systems. Note: no IL-10 was detected in the trans-well system (B). 0 hr  =  serum starved adipocytes alone prior to co-culture and fatty acid treatment, (−)  =  negative control; adipocytes alone treated with 25 µM BSA, (+)  =  positive control; co-cultured adipocytes and macrophages plus 25 µM BSA, DHA =  co-cultured adipocytes and macrophages in the presence of 125 µM DHA, EPA =  co-cultured adipocytes and macrophages in the presence of 125 µM EPA, and PA =  co-cultured adipocytes and macrophages in the presence of 125 µM PA. Values are means ± SEM. The experiment was independently conducted 3 times for a final sample size of n =  6–9. A different letter or an asterisk (*) indicates treatments are significantly different from each other, p≤0.05.

Secreted adipokines (MCP1, IL-6, TNFα and IL-10) were measured in the culture supernatant in both the contact (**Figure 1A**) and trans-well (**Figure 1B**) co-culture systems. In the contact and trans-well co-culture systems the secretion of pro-inflammatory adipokines were increased when adipocytes were cultured with macrophages only (positive control) compared to the negative control (adipocytes alone treated with BSA), in contact (+327% MCP1, +147% IL-6), and trans-well (+101% MCP1, +817% TNFα, p≤0.05), thereby demonstrating that pro-inflammatory cross-talk occurs between these cell types. Interestingly, significant increases in secreted IL-10 (p≤0.05, **Figure 1A**) relative to 0 hr were dependant on macrophage contact since no IL-10 was detectable in the trans-well system (data not shown). Similarly, in all co-culture conditions, secreted MCP1 and IL-6 in the contact system was at least 1.5-fold higher than the level detected in the trans-well system under the same culture conditions/treatment (p≤0.05, **Figure 1C**). However, the reverse trend was seen for secreted TNFα in the positive control; there was a 2.0-fold increase in TNFα secretion in the trans-well system relative to the contact system (p≤0.05, **Figure 1C**).

### Fatty acids differentially modulate MCP1, IL-6 and TNFα secretion in both the contact and trans-well co-culture systems

MCP1 secretion was reduced by DHA (−57% contact, −46% trans-well, p≤0.05) and EPA (−48% contact, −27% trans-well, p≤0.05) compared to the positive control (adipocytes plus macrophages only, no fatty acid treatment) in both the contact (**Figure 1A**) and trans-well (**Figure 1B**) co-culture systems. Interestingly, DHA decreased IL-6 secretion (−63% contact, −41% trans-well, p≤0.05) compared to the positive control, while EPA did not (**Figure 1A & B**). Moreover, only EPA increased TNFα secretion in the contact system relative to the negative control (+471%, p≤0.05, **Figure 1A**), whereas both DHA and EPA decreased TNFα secretion to a similar extent relative to the positive control in the trans-well system (−46%, p≤0.05, **Figure 1B**). Overall, PA was largely no more pro-inflammatory than the positive control, as assessed by MCP1, TNFα and IL-6 secretion, with the exception of a small reduction in MCP1 secretion (−14%, p≤0.05) in the trans-well system (**Figure 1B**).

### Fatty acids do not affect cellular cytokine protein concentrations in co-cultured adipocytes in the trans-well system

Adipocyte cellular protein could only be collected from cells in the trans-well culture system and the cellular protein of all cytokines assessed were increased (p≤0.05) at 12 hr compared to pre-treatment (0 hr) without the addition of BSA (negative control) or macrophages (positive control; **Figure S1**). However, cellular adipocyte IL-6, IL-10, TNFα and MCP1 protein was not affected by any treatment (**Figure S1**).

### Fatty acids differentially modulate adipokine and inflammatory mediator mRNA expression in co-cultured adipocytes in the trans-well system

**Figure 2:**
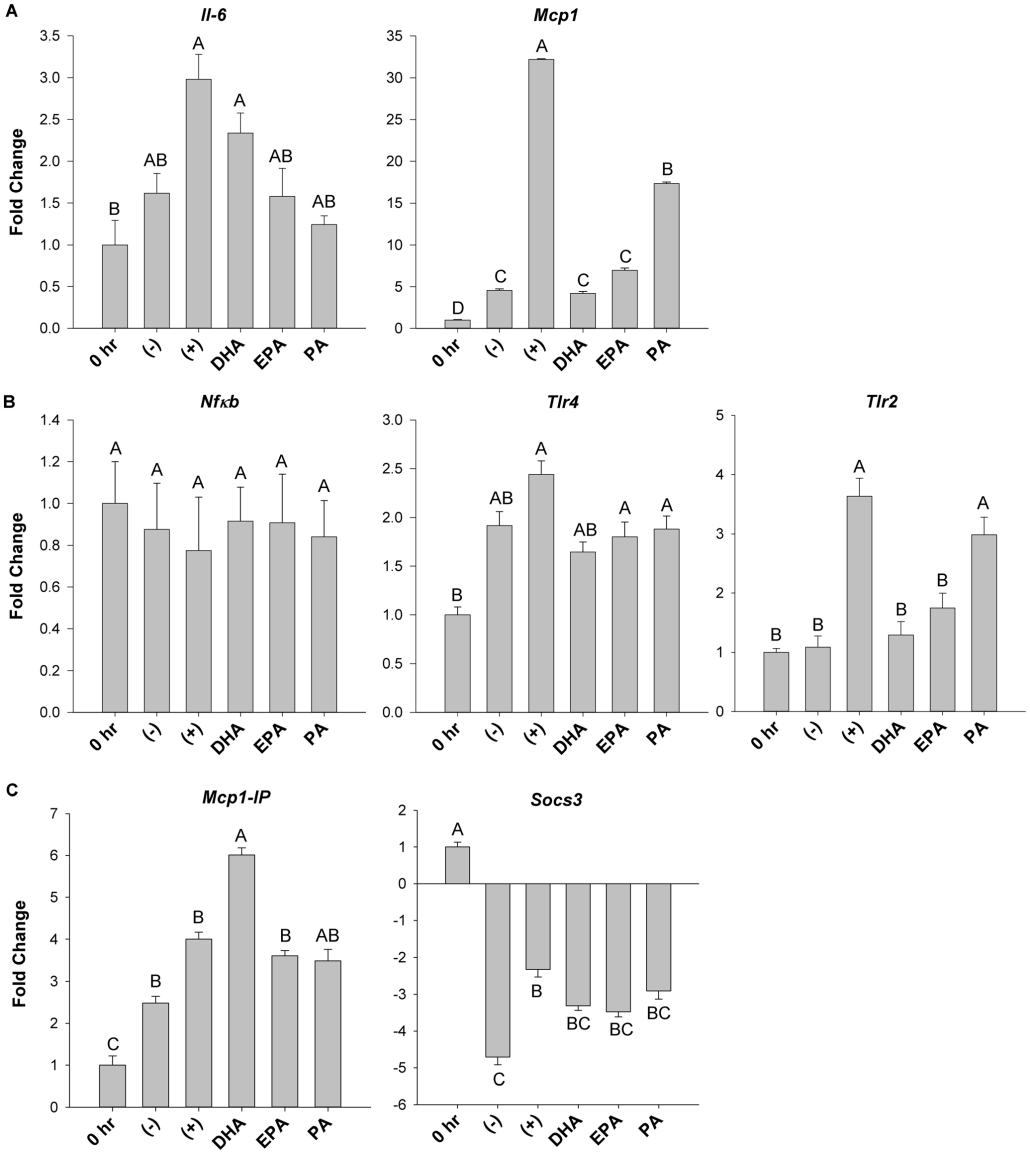
mRNA expression of inflammatory mediators in trans-well co-cultured adipocytes. The mRNA expression of key inflammatory (A) cytokines (*Il-6* and *Mcp1*), (B) signalling intermediates (*Nfκb*, *Tlr4* and *Tlr2*) and (C) negative feedback factors (*Mcp1-IP* and *Socs3*) from adipocytes harvested from the trans-well system after 12 hr of co-culture. 0 hr  =  serum starved adipocytes alone prior to co-culture and fatty acid treatment, (−)  =  negative control; adipocytes alone treated with 25 µM BSA, (+)  =  positive control; co-cultured adipocytes and macrophages plus 25 µM BSA, DHA  =  co-cultured adipocytes and macrophages in the presence of 125 µM DHA, EPA =  co-cultured adipocytes and macrophages in the presence of 125 µM EPA, and PA =  co-cultured adipocytes and macrophages in the presence of 125 µM PA. Values are mean fold change ± SEM. The experiment was independently conducted 3 times (in triplicate) for a final sample size of n = 6–9. A different letter indicates treatments are significantly different from each other, p≤0.05.

Since the trans-well system allowed for isolation of RNA from each cell type used in co-culture (adipocytes and macrophages), mRNA expression of critical inflammatory adipokines (*Il-6*, *Mcp1*), signalling intermediates (*Nfκb*, *Tlr4*, *Tlr2*) and negative feedback factors (*Mcp1-IP, Socs3*) was assessed in adipocytes exposed to the various treatments. mRNA expression of *Il-6* in fatty acid treatment groups was not significantly decreased relative to the positive control (adipocytes plus macrophages only, **Figure 2A**). In contrast, relative to the positive control, mRNA expression of *Mcp1* was decreased by DHA (−7.1 fold, p≤0.05), EPA (−4.9 fold, p≤0.05) and PA (−2.1 fold, p≤0.05). Moreover, both DHA (−3.9 fold, p≤0.05) and EPA (−2.7 fold, p≤0.05) further reduced *Mcp1* mRNA expression compared to PA (**Figure 2A**). Of note, adipocyte mRNA expression of *Il-10* and *Tnfα* was negligible under all treatment conditions in the trans-well system (data not shown). Similarly, adipocyte mRNA expression of *Nfκb* was not affected by fatty acid treatment (**Figure 2B**). Furthermore, only DHA prevented the increase (+2.3 fold, p≤0.05) in *Tlr4* expression (**Figure 2B**) induced by the positive control relative to the pre-treatment at 0 hr. In contrast, mRNA expression of *Tlr2* was down-regulated by both DHA (−2.9 fold, p≤0.05) and EPA (−2.3 fold, p≤0.05) relative to the positive control and PA (−2.5 fold and −1.8 fold, DHA and EPA, respectively, p≤0.05 **Figure 2B**). Intriguingly, relative to the positive control, only DHA up-regulated mRNA expression of *Mcp1-IP* (+1.5 fold, p≤0.05, **Figure 2C**), a negative regulator of MCP1 signalling. In contrast, co-culture of macrophages plus adipocytes (positive control) led to an increase in mRNA expression of a negative regulator of IL-6, *Socs3* (+1.9 fold, p≤0.05) relative to the negative control (adipocytes alone plus BSA), whereas other fatty acids did not (**Figure 2C**). However, *Socs3* mRNA expression did not differ between fatty acid treatments (p>0.05).

### NFκB activity is not affected by fatty acid treatments in trans-well co-cultured macrophages or adipocytes

**Figure 3:**
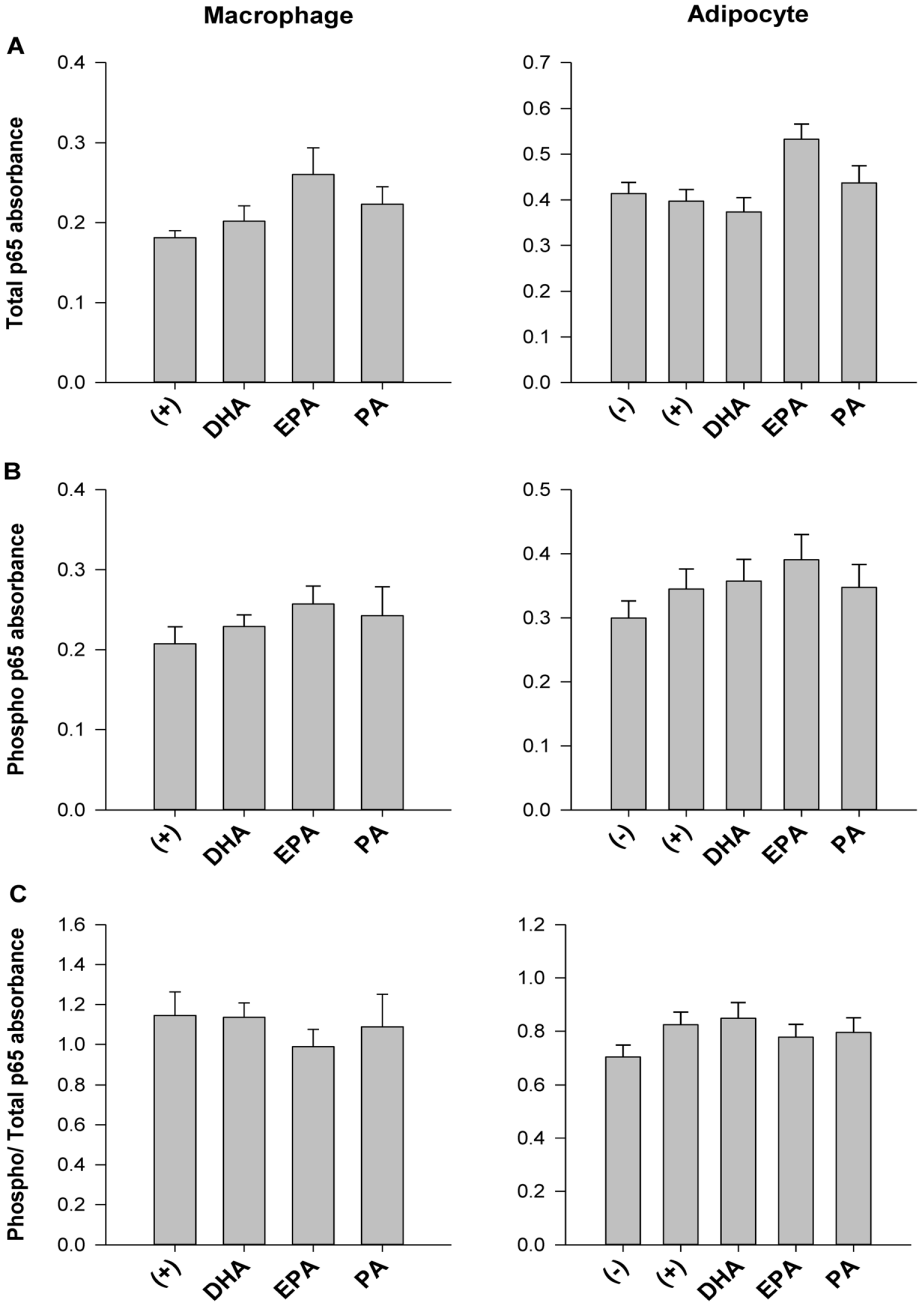
NFκB activity in trans-well co-cultured adipocytes. The absorbance (at 450 nm) of (A) total p65 NFκB and (B) phosphorylated- p65 NFκB, and (C) the ratio of phosphorylated- p65 NFκB: total p65 NFκB as a measure of NFκB activity in adipocytes and macrophages harvested from the trans-well system at 12 hr. 0 hr  =  serum starved adipocytes alone prior to co-culture and fatty acid treatment, (−)  =  negative control; adipocytes alone treated with 25 µM BSA, (+)  =  positive control; co-cultured adipocytes and macrophages plus 25 µM BSA, DHA  =  co-cultured adipocytes and macrophages in the presence of 125 µM DHA, EPA =  co-cultured adipocytes and macrophages in the presence of 125 µM EPA, and PA =  co-cultured adipocytes and macrophages in the presence of 125 µM PA. Values are mean fold change ± SEM. The experiment was independently conducted 2 times (in triplicate) for a final sample size of n = 6. A different letter indicates treatments are significantly different from each other, p≤0.05.

Both total (**Figure 3A**) and phosphorylated (i.e. activated) p65 NFκB (**Figure 3B**) were similar between treatments (p>0.05) in trans-well co-cultured adipocytes and macrophages. Additionally, NFκB activity, i.e. the ratio of phosphorylated/total p65 NFκB, was similar across treatments in trans-well co-cultured adipocytes and macrophages, regardless of fatty acid treatment (p>0.05; **Figure 3C**).

### DHA decreases mRNA expression of key M1 and M2 polarization markers but increases mRNA expression of regulatory cytokines Tgfβ1 and IL-10 in macrophages co-cultured in the trans-well system

**Figure 4:**
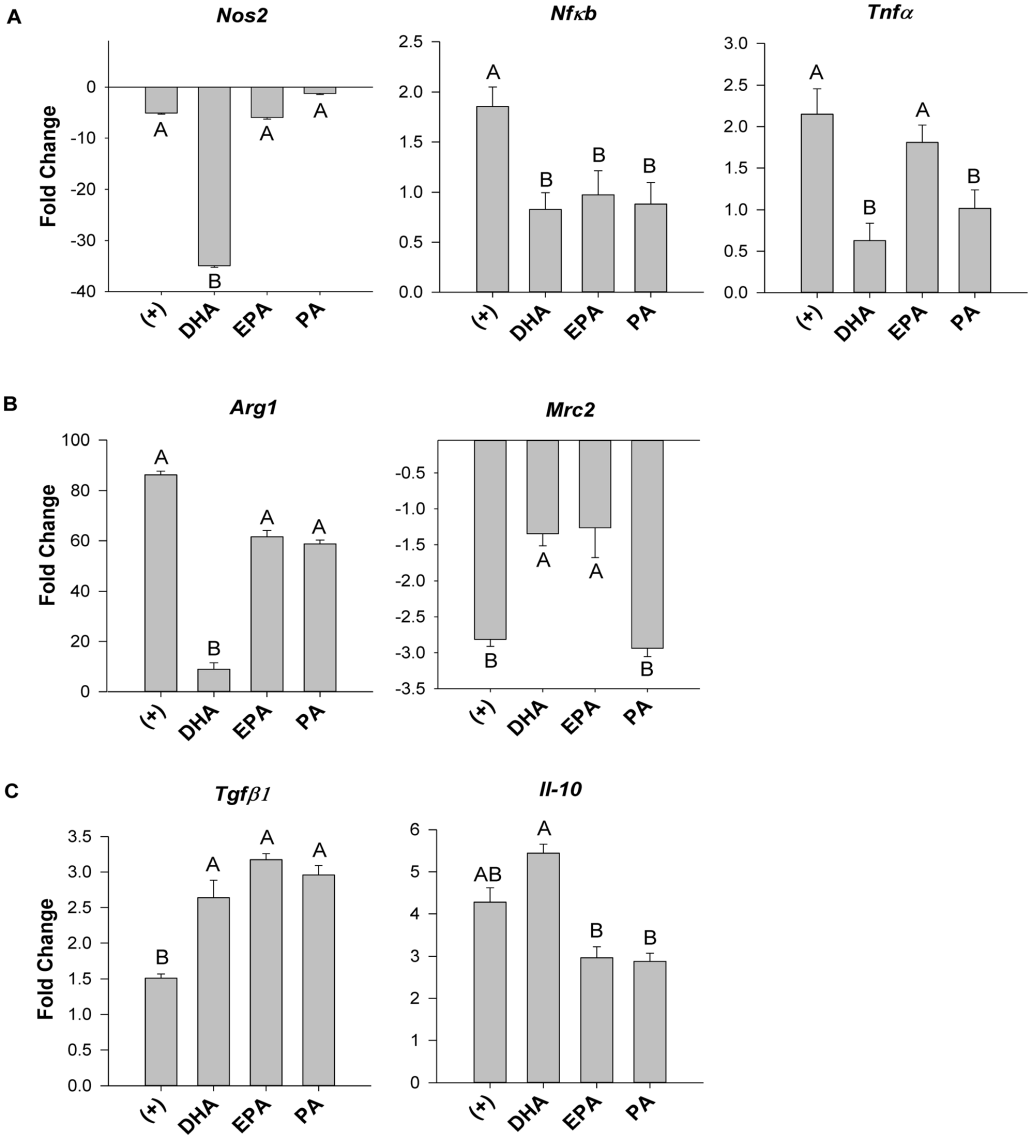
M1 and M2 macrophage polarization marker mRNA expression in trans-well co-cultured macrophages. mRNA expression of key (A) M1 (*Nos2*, *Nfκb* and *Tnfα*), (B) M2 (*Arg1*, *Mrc2*) polarization genes, and (C) regulatory cytokines (*Tgfβ1* and *Il-10*) from macrophages harvested from the trans-well system at 12 hr. 0 hr  =  serum starved adipocytes alone prior to co-culture and fatty acid treatment, (+)  =  positive control; co-cultured adipocytes and macrophages plus 25 µM BSA, DHA  =  co-cultured adipocytes and macrophages in the presence of 125 µM DHA, EPA =  co-cultured adipocytes and macrophages in the presence of 125 µM EPA, and PA =  co-cultured adipocytes and macrophages in the presence of 125 µM PA. Values are mean fold change ± SEM. The experiment was independently conducted 2 times (in triplicate) for a final sample size of n = 6. A different letter indicates treatments are significantly different from each other, p≤0.05.

Macrophage specific mRNA expression of M1 (*Nos2*, *Nfκb* and *Tnfα*), M2 (*Arg1, Mrc2*) polarization markers and regulatory cytokines (*Tgfβ1* and *Il-10*) were assessed from cells co-cultured in the trans-well system under all fatty acid treatment conditions. The addition of DHA to the trans-well co-culture system resulted in decreased macrophage mRNA expression of key M1 polarization markers including: *Nos2* (−7 fold, p≤0.05), *Nfκb* (−2.3 fold, p≤0.05) and *Tnfα* (−4.2 fold, p≤0.05) relative to co-culture of adipocytes plus macrophages only (positive control; **Figure 4A**). Other fatty acids were not as potent as DHA, but still exerted some significant effects, namely, EPA-treated macrophages reduced *Nfκb* expression (−1.9 fold, p≤0.05), and PA decreased mRNA expression of *Nfκb* (−2.1 fold, p≤0.05) and *Tnfα* (−2.2 fold, p≤0.05) relative to the positive control (**Figure 4A**). Macrophage mRNA expression of the M2 polarization marker *Arg1* was reduced by DHA treatment (−8.5 fold, p≤0.05) (**Figure 4B**). Additionally, all co-culture conditions decreased mRNA expression of the mannose receptor *Mrc2*, another M2 polarization marker; however, the magnitude of this effect was partially reversed by both DHA and EPA (by approximately +2.0 fold, p≤0.05) relative to both the positive control and PA (**Figure 4B**). With regard to anti-inflammatory regulatory cytokine expression, *Tgfβ1* mRNA expression was increased by all fatty acid treatments (p≤0.05) relative to the positive control (**Figure 4C**). Conversely, DHA increased mRNA expression of *Il-10*, relative to EPA and PA (+1.8 fold, p≤0.05), and exhibited a trend towards increased expression (+1.3 fold, p<0.07) relative to the positive control (**Figure 4C**).

### Acute PPARγ antagonism affects adiponectin cellular protein, but not other cytokine cellular protein or mRNA expression in trans-well co-cultured adipocytes

**Figure 5:**
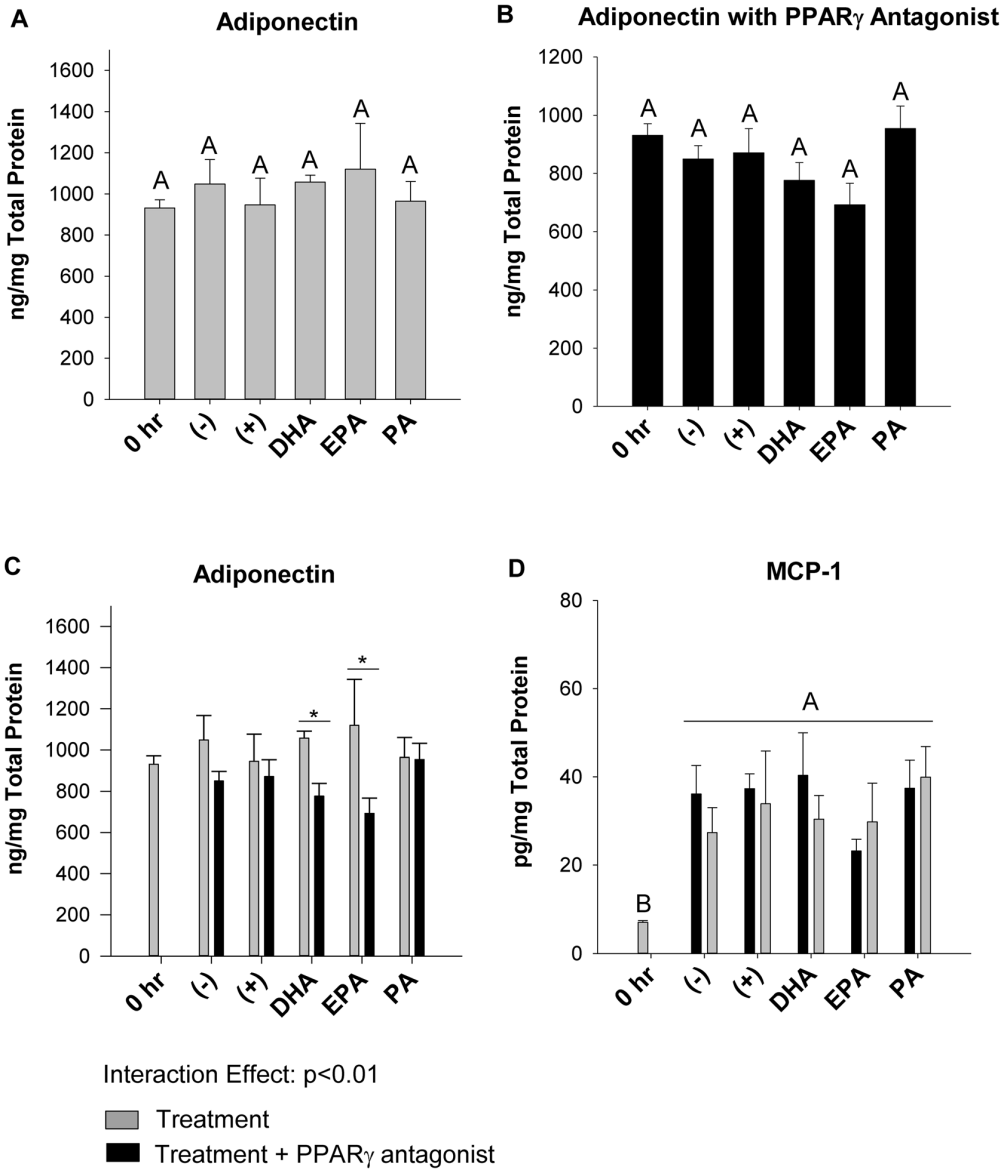
Effect of a Pparγ antagonist on adiponectin and MCP1 cellular protein in trans-well co-cultured adipocytes. The cellular protein concentrations of adiponectin harvested from adipocytes the trans-well system at 12 hr alone (A) or with the PPARγ antagonist, T0070907 added (B), and a comparison of adiponectin (C) or MCP1 (D) cellular protein with and without the PPARγ antagonist added. 0 hr  =  serum starved adipocytes alone prior to co-culture and fatty acid treatment, (−)  =  negative control; adipocytes alone treated with 25 µM BSA, (+)  =  positive control; co-cultured adipocytes and macrophages plus 25 µM BSA, DHA  =  co-cultured adipocytes and macrophages in the presence of 125 µM DHA, EPA =  co-cultured adipocytes and macrophages in the presence of 125 µM EPA, and PA =  co-cultured adipocytes and macrophages in the presence of 125 µM PA. Values are means ± SEM. The experiment was independently conducted 2 times (in triplicate) for a final sample size of n = 6. A different letter or an asterisk (*) indicates treatments are significantly different from each other, p≤0.05.

Adding the PPARγ antagonist did not affect MCP1, IL-6, TNFα or IL-10 mRNA expression, cellular or secreted protein in adipocytes co-cultured in the trans-well system relative to treatment conditions without the antagonist (representative data shown in **Figure 5**). Additionally, the PPARγ antagonist had no effect on macrophage polarization marker mRNA expression in trans-well co-cultured macrophages (data not shown). Interestingly, with or without the PPARγ antagonist, adiponectin protein was not significantly affected by any treatment (**Figure 5A and B**). However, when the PPARγ antagonist was present in the trans-well system, adiponectin cellular protein was decreased (p≤0.05) in co-cultured adipocytes in DHA or EPA-treated cultures (−27% and −38%, respectively, **Figure 5C**). In contrast, cellular MCP1 protein in adipocytes was not affected by the presence of the PPARγantagonist (p>0.05, **Figure 5D**).

## Discussion

In obesity, paracrine interactions between adipocytes and adipose-infiltrating macrophages contribute to chronic inflammation characterized by abnormal secretion of pro-inflammatory adipokines [1], [2]. Since long-chain n-3 PUFA like DHA and EPA can exert anti-inflammatory effects [14], [15], they may represent a dietary strategy to mitigate the harmful effects of pro-inflammatory adipokines. In the current study, we showed for the first time that co-culturing adipocytes and macrophages at a ratio of macrophages to adipocytes that is representative of obese adipose tissue [22], either in direct contact (contact-dependent mechanism) or separated by a porous trans-well insert (contact-independent mechanism), promoted a pro-inflammatory adipokine profile with elevations in secreted MCP1, IL-6 and TNFα (**Figure 1**). Interestingly, adipocytes and macrophages co-cultured in direct contact led to a relative doubling of secreted adipokines (with the exception of TNFα) as compared to the trans-well system, suggesting that direct cell to cell contact, and not just the presence of soluble mediators, preferentially drives pro-inflammatory cross-talk between adipocytes and macrophages (**Figure 1C**). This trend has been shown elsewhere with IL-6 secretion in trans-well and contact co-culture conditions [25], but our study extends previous findings [3], [26] and, to our knowledge, is the first to demonstrate that cell co-culture conditions (contact-dependent versus contact-independent), in addition to the presence of DHA and EPA, modifies the secreted adipokine profile. Furthermore, we showed for the first time in adipocyte:macrophage co-culture conditions without a potent pro-inflammatory stimulus (e.g. lipopolysaccharide) or pre-co-culture incubation with n-3 PUFA [26], that DHA decreased mRNA expression of M1 (*Tnfα* and *Nos2*) and M2 associated genes (*Mrc2* and *Arg1*), while simultaneously increasing mRNA expression of the regulatory and anti-inflammatory cytokines, *Tgfβ1* and *Il-10* in co-culture (**Figure 4**). Our findings support the idea that a possible mixed macrophage phenotype may exist in obese adipose tissue, characterized by expressing various levels of both M1 and M2 polarization markers [27], [28], however, additional characterization of the phenotype beyond gene expression of such markers (e.g. by flow cytometry) requires further study to confirm our findings. Finally, we showed that the selective PPARγ antagonist, T0070907, reduced adiponectin cellular protein in co-cultured adipocytes from DHA and EPA treated cultures (**Figure 5C**), without affecting other cytokine mRNA, cellular or secreted protein concentrations in this co-culture model (representative data in **Figure 5D**), thereby supporting the idea that antagonists that impair PPARγ transcriptional activity may not always promote pro-inflammatory responses [29], [30].

Analysis of secreted adipokine protein (**Figure 1**) and mRNA expression (**Figure 2**) in co-cultured adipocytes revealed further mechanistic insight into how DHA and EPA modulate inflammation in co-culture. DHA had the most potent anti-inflammatory effect as evidenced by a significant reduction in secreted MCP1 and IL-6 in both co-culture conditions. While EPA had somewhat similar effects to DHA in our model, it was unable to suppress the secretion of IL-6. These findings are consistent with a recent study showing that DHA can exert a more potent anti-inflammatory effect compared to EPA in co-culture [26]. Interestingly, while n-3 PUFA exerted similar effects on both *Mcp1* mRNA expression in adipocytes and secreted protein, this trend was not observed with respect to *Il-6*. Unexpectedly, PA resulted in a more potent decrease in *Il-6* mRNA expression in adipocytes compared with DHA and EPA. However, the 12 hr incubation used in our co-culture experiments may have been too short to see elevations in adipocyte *Il-6* mRNA expression since, in 3T3-L1 adipocytes alone, 125 µM of PA has been shown to increase *Il-6* mRNA expression after 24 hr [5]. Although the adipocyte *Il-6* mRNA expression profile we observed was similar to what has previously been observed in co-culture [3], we did not see a PA-induced up-regulation of the classic IL-6 negative regulator, *Socs3*. Additionally, PA did not up-regulate mRNA expression of the negative regulator, *Mcp1-IP* in adipocytes compared to the positive control (adipocytes plus macrophages only), therefore it does not appear that MCP1-IP, which can act as an RNase to degrade *Il-6* mRNA expression [8], was playing a key role in the PA co-culture treatment. In contrast, this negative feedback system involving MCP1-IP may have been induced by DHA in co-culture (**Figure 2C**); however, the degree of DHA's anti-inflammatory action elicited through MCP1-IP requires further investigation. Moreover, we observed that DHA and EPA reduce *Tlr2* mRNA expression in co-cultured adipocytes, suggesting that these fatty acids more potently regulate *Tlr2* expression than *Tlr4*. Indeed, TLR2 has been shown to be a key signalling pathway that EPA targets to downregulate *Il-6* mRNA expression in adipose tissue stem cells [31], therefore, the TLR2 pathway also warrants further study in co-culture conditions. We did not detect substantial fatty acid-induced differences in adipocyte *Nfκb* or *Tlr4* expression in our model. Furthermore, we observed that co-culture conditions (regardless of fatty acid treatment) did not increase NFκB activity, i.e. the ratio of phosphorylated/total p65 NFκB in trans-well co-cultured adipocytes or macrophages (**Figure 3**), suggesting that measuring phosphorylated p65 NFκB (i.e. RelA; the transcriptionally active NFκB sub-unit), may not be a useful endpoint to measure the inflammatory status of cells after 12 hr co-culture incubations. Finally, additional measurement of inflammatory signalling intermediates (e.g. JNK, MAPK, STAT3 etc.) is warranted in co-culture conditions to delineate if fatty acid treatments differentially modulate signalling through these inflammatory pathways in adipocytes or macrophages.

One of the challenges to mitigating chronic inflammation is decreasing the degree of M1-like macrophage polarization in macrophages infiltrated into obese adipose tissue, while increasing anti-inflammatory M2-like macrophages that secrete IL-10 and help resolve inflammation [32]. In our macrophage adipocyte co-culture model we examined the role of fatty acids in altering key inflammatory mediators implicated in the M1 (*Nos2*, *Nfκb*, *Tnfα*) and M2 (*Arg1*, *Mrc2*, *Tgfβ1*, *IL-10*) macrophage polarization response. Similar to the effect of fatty acids on the secreted adipokine milieu, DHA was more anti-inflammatory than EPA, as evidenced by decreased mRNA expression of M1 markers *Tnfα*, iNOS (*Nos2*), and *Nfκb*, which are associated with the pro-inflammatory M1 macrophage phenotype. These phenomena may be partly attributed to the anti-inflammatory actions of PPARγ, including trans-repression of NFκB [33], since monocytes express PPARγ [34] and DHA is a potent PPARγ agonist [35]. Interestingly, EPA and PA also decreased *Nfκb* mRNA expression to a similar degree as DHA. Since EPA [35] and even PA [36] to some extent may also act as PPARγ agonists, it is possible that trans-repression could occur here too, although this requires further investigation.

Collectively, the mRNA expression data in our model suggests that DHA-treated macrophages in co-culture with adipocytes display characteristics that are consistent with promoting a regulatory macrophage phenotype. The presence of such regulatory macrophages in obese adipose tissue may be ideal given their secretion of the anti-inflammatory cytokines, IL-10 and TGFβ1, and the lack of antigen presentation to T helper cells [32] that could exacerbate chronic inflammation in obesity [37]. Finally, it is known that adiponectin may promote a regulatory macrophage phenotype *in vitro* and *ex vivo* [20], [21], and that DHA supplementation in a rodent high-fat diet induced obesity model promotes M2 macrophage polarization in adipose tissue [38]. Therefore, further research into the role of fatty acids in macrophage polarization is needed, particularly by PPARγ agonists, such as DHA, that promote adiponectin production after 24 hr [18], [19].

Since previous studies have shown the involvement of PPARγ in DHA and EPA-mediated effects [18], [19], we performed experiments using the selective PPARγ antagonist, T0070907. Interestingly, in our model the addition of the PPARγ antagonist in co-culture with adipocytes and macrophages plus DHA or EPA decreased adiponectin cellular protein concentrations (**Figure 5C**), without affecting other inflammatory mediators and cytokines (representative data in **Figure 5D**). These findings initially seemed counterintuitive since PPARγ can exert anti-inflammatory effects [33], [39], and plays an essential role in IL-4-induced M2 macrophage polarization [40]. However, these anti-inflammatory effects may be context-dependant as PPARγ agonists, such as thiazolidinediones, are not always anti-inflammatory [41], and the use of PPARγ antagonists does not always promote a pro-inflammatory response [29], [30]. Additionally, PPARγ agonists (e.g. rosiglitazone) and non-agonist ligands (e.g. SR1664; [42]) may decrease inflammatory signalling and preserve insulin sensitivity through non-canonical PPARγ signalling independent of transcriptional regulation (reviewed by [43]). Overall, our data suggests that a more careful evaluation of PPARγ antagonists is needed before we can use them in experimental models to isolate the PPARγ-independent anti-inflammatory activities of n-3 PUFA.

In setting up this study we made decisions regarding fatty acid dose and incubation time that require further explanation. Firstly, previous findings determined that 125 µM DHA maximally increased secreted adiponectin in 3T3-L1 adipocytes [18] and therefore, we utilized this dosage for all fatty acids. Interestingly, 125 µM of PA did not induce additional pro-inflammatory cytokine secretion in co-culture relative to adipocytes and macrophages only (positive control). Although PA is thought to be pro-inflammatory due to its ability to act as a TLR4 agonist [4], [6], [44], and its ability to increase reactive oxygen species production and subsequent MCP1 secretion in adipocytes [30], [45], these effects may only be evident at higher doses (200–500 µM) and incubations longer than 12 hr [4], [5], [30]. Secondly, with regards to the timing of fatty acid exposure, we found fatty acid-induced differences in secreted cytokines (e.g. IL-6 and MCP1) after 12 hr, which may have been too short to see changes in other secreted cytokines, such as IL-10, as previous reports have shown that DHA increases IL-10 secretion in 3T3-L1 adipocytes after 24 hr [46].

In summary, our results demonstrate that fatty acids modulate the pro-inflammatory adipokine milieu generated in a co-culture model designed to represent the ratio of macrophages to adipocytes seen in obese adipose tissue [22]. Overall, this data suggests that macrophage presence is necessary to induce pro-inflammatory cross-talk with adipocytes. Moreover, our study suggests that DHA may act to suppress inflammation concomitantly in both cell types. For one, DHA decreased the degree of M1 polarization marker mRNA expression in macrophages while increasing expression of the potent anti-inflammatory cytokines, *Tgfβ1* and *Il-10*, thereby driving a gene expression profile that is consistent with promoting a regulatory macrophage phenotype. This data suggests that DHA may suppress macrophage activation in co-culture, which could then feedback to inhibit inflammatory cytokine release (e.g. MCP1 and IL-6) from adipocytes since DHA, and to a lesser extent EPA, decreased MCP1 and IL-6 secretion and *Mcp1* mRNA expression in co-cultured adipocytes. Taken together, n-3 PUFA could decrease the intensity of pro-inflammatory cross-talk between adipocytes and macrophages, which may partly explain the decreased macrophage infiltration into obese adipose tissue observed in some rodent models [17]. Thus, dietary n-3 PUFA, in particular DHA, may be a useful strategy to mitigate the effects of obesity-associated inflammation.

## Supporting Information

Figure S1 **Adipocyte cellular protein in the trans-well system.** The cellular protein concentrations of key cytokines (IL-6, MCP-1, TNFα and IL-10) measured from adipocytes in the trans-well system at 12 hr. 0 hr  =  serum starved adipocytes alone prior to co-culture and fatty acid treatment, (−)  =  negative control; adipocytes alone treated with 25 µM BSA, (+)  =  positive control; co-cultured adipocytes and macrophages plus 25 µM BSA, DHA  =  co-cultured adipocytes and macrophages in the presence of 125 µM DHA, EPA =  co-cultured adipocytes and macrophages in the presence of 125 µM EPA, and PA =  co-cultured adipocytes and macrophages in the presence of 125 µM PA. Values are means ± SEM. The experiment was independently conducted 2 times (in triplicate) for a final sample size of n =  6. A different letter indicates treatments are significantly different from each other, p≤0.05.
(PDF)

## References

[1] Galic S, Oakhill JS, Steinberg GR (2010) Adipose tissue as an endocrine organ. Mol Cell Endocrinol 316: 129–139. 19723556 10.1016/j.mce.2009.08.018

[2] Surmi BK, Hasty AH (2008) Macrophage infiltration into adipose tissue: Initiation, propagation and remodeling. Future lipidol 3: 545–556. 18978945 10.2217/17460875.3.5.545 PMC2575346

[3] Suganami T, Nishida J, Ogawa Y (2005) A paracrine loop between adipocytes and macrophages aggravates inflammatory changes role of free fatty acids and tumor necrosis factor α. Arterioscler Thromb Vasc Biol 25: 2062–2068. 16123319 10.1161/01.ATV.0000183883.72263.13

[4] Suganami T, Tanimoto-Koyama K, Nishida J, Itoh M, Yuan X, et al (2007) Role of the toll-like receptor 4/NF-κB pathway in saturated fatty acid–induced inflammatory changes in the interaction between adipocytes and macrophages. Arterioscler Thromb Vasc Biol 27: 84–91. 17082484 10.1161/01.ATV.0000251608.09329.9a

[5] Ajuwon KM, Spurlock ME (2005) Palmitate activates the NF-kappaB transcription factor and induces IL-6 and TNFalpha expression in 3T3-L1 adipocytes. J Nutr 135: 1841–1846. 16046706 10.1093/jn/135.8.1841

[6] Lee JY, Plakidas A, Lee WH, Heikkinen A, Chanmugam P, et al (2003) Differential modulation of toll-like receptors by fatty acids preferential inhibition by n-3 polyunsaturated fatty acids. J Lipid Res 44: 479–486. 12562875 10.1194/jlr.M200361-JLR200

[7] Narazaki M, Fujimoto M, Matsumoto T, Morita Y, Saito H, et al (1998) Three distinct domains of SSI-1/SOCS-1/JAB protein are required for its suppression of interleukin 6 signalling. Proc Natl Acad Sci USA 95: 13130–13134. 9789053 10.1073/pnas.95.22.13130 PMC23734

[8] Matsushita K, Takeuchi O, Standley DM, Kumagai Y, Kawagoe T, et al (2009) Zc3h12a is an RNase essential for controlling immune responses by regulating mRNA decay. Nature 458: 1185–1190. 19322177 10.1038/nature07924

[9] Scheele C, Nielsen S, Kelly M, Broholm C, Nielsen AR, et al (2012) Satellite cells derived from obese humans with type 2 diabetes and differentiated into myocytes in vitro exhibit abnormal response to IL-6. PLoS One 7: e39657. 22761857 10.1371/journal.pone.0039657 PMC3383673

[10] Lumeng CN, DeYoung SM, Bodzin JL, Saltiel AR (2007) Increased inflammatory properties of adipose tissue macrophages recruited during diet-induced obesity. Diabetes 56: 16–23. 17192460 10.2337/db06-1076

[11] Lumeng CN, Bodzin JL, Saltiel AR (2007) Obesity induces a phenotypic switch in adipose tissue macrophage polarization. J Clin Invest 117: 175–184. 17200717 10.1172/JCI29881 PMC1716210

[12] Lumeng CN, Deyoung SM, Saltiel AR (2007) Macrophages block insulin action in adipocytes by altering expression of signalling and glucose transport proteins. Am J Physiol Endocrinol Metab292: E166–E174. 10.1152/ajpendo.00284.2006 PMC3888778 16926380

[13] Abel ED, Peroni O, Kim JK, Kim Y, Boss O (2001) Adipose-selective targeting of the GLUT4 gene impairs insulin action in muscle and liver. Nature 409: 729–733. 11217863 10.1038/35055575

[14] Calder PC (2006) n-3 polyunsaturated fatty acids, inflammation, and inflammatory diseases. Am J Clin Nutr 83: S1505–1519S. 10.1093/ajcn/83.6.1505S 16841861

[15] Turk HF, Chapkin RS (2012) Membrane lipid raft organization is uniquely modified by n-3 polyunsaturated fatty acids. Prostaglandins, Leukot Essent Fatty Acids 88: 43–47. 22515942 10.1016/j.plefa.2012.03.008 PMC3404206

[16] Flachs P, Mohamed-Ali V, Horakova O, Rossmeisl M, Hosseinzadeh-Attar M, et al (2006) Polyunsaturated fatty acids of marine origin induce adiponectin in mice fed a high-fat diet. Diabetologia 49: 394–397. 16397791 10.1007/s00125-005-0053-y

[17] Todoric J, Löffler M, Huber J, Bilban M, Reimers M, et al (2006) Adipose tissue inflammation induced by high-fat diet in obese diabetic mice is prevented by n-3 polyunsaturated fatty acids. Diabetologia 49: 2109–2119. 16783472 10.1007/s00125-006-0300-x

[18] Oster RT, Tishinsky JM, Yuan Z, Robinson LE (2010) Docosahexaenoic acid increases cellular adiponectin mRNA and secreted adiponectin protein, as well as PPARγ mRNA, in 3T3-L1 adipocytes. Appl Physiol Nutr Metab35: 783–789. 10.1139/H10-076 21164549

[19] Tishinsky JM, Ma DW, Robinson LE (2010) Eicosapentaenoic acid and rosiglitazone increase adiponectin in an additive and PPARγ-dependent manner in human adipocytes. Obesity 19: 262–268. 20814411 10.1038/oby.2010.186

[20] Park P, McMullen MR, Huang H, Thakur V, Nagy LE (2007) Short-term treatment of RAW264.7 macrophages with adiponectin increases tumor necrosis factor-α (TNF-α) expression via ERK1/2 activation and egr-1 expression: role of TNF-α in adiponectin-stimulated interleukin-10 production. J Biol Chem 282: 21695–21703. 17537727 10.1074/jbc.M701419200 PMC1978175

[21] Mandal P, Pratt BT, Barnes M, McMullen MR, Nagy LE (2011) Molecular mechanism for adiponectin-dependent M2 macrophage polarization link between the metabolic and innate immune activity of full-length adiponectin. J Biol Chem 286: 13460–13469. 21357416 10.1074/jbc.M110.204644 PMC3075692

[22] Kanda H, Tateya S, Tamori Y, Kotani K, Hiasa K, et al (2006) MCP1 contributes to macrophage infiltration into adipose tissue, insulin resistance, and hepatic steatosis in obesity. J Clin Invest 116: 1494–1505. 16691291 10.1172/JCI26498 PMC1459069

[23] Wilson S, Wong M, Stryjecki C, De Boer A, Lui E, et al (2013) Unraveling the adipocyte inflammomodulatory pathways activated by North American ginseng. Int J Obes (Lond) 37: 350–356. 22508335 10.1038/ijo.2012.50

[24] Lee G, Elwood F, McNally J, Weiszmann J, Lindstrom M, et al (2002) T0070907, a selective ligand for peroxisome proliferator-activated receptor γ, functions as an antagonist of biochemical and cellular activities. J Biol Chem 277: 19649–19657. 11877444 10.1074/jbc.M200743200

[25] Xie L, Ortega MT, Mora S, Chapes SK (2010) Interactive changes between macrophages and adipocytes. Clin Vaccine Immunol 17: 651–659. 20164250 10.1128/CVI.00494-09 PMC2849320

[26] Oliver E, McGillicuddy FC, Harford KA, Reynolds CM, Phillips CM, et al (2012) Docosahexaenoic acid attenuates macrophage-induced inflammation and improves insulin sensitivity in adipocytes-specific differential effects between LC n-3 PUFA. J Nutr Biochem 23: 1192–1200. 22137266 10.1016/j.jnutbio.2011.06.014

[27] Shaul ME, Bennett G, Strissel KJ, Greenberg AS, Obin MS (2010) Dynamic, M2-like remodeling phenotypes of CD11c adipose tissue macrophages during high-fat diet–induced obesity in mice. Diabetes 59: 1171–1181. 20185806 10.2337/db09-1402 PMC2857897

[28] Sica A, Mantovani A (2012) Macrophage plasticity and polarization: In vivo veritas. J Clin Invest 122: 787–795. 22378047 10.1172/JCI59643 PMC3287223

[29] Chen C, Chang Y, Tsi C, Lin W (2003) Inhibition of IFN-γ-mediated inducible nitric oxide synthase induction by the peroxisome proliferator-activated receptor γ agonist, 15-deoxy-Δ12, 14-prostaglandin J2, involves inhibition of the upstream janus kinase/STAT1 signalling pathway. J Immunol 171: 979–988. 12847270 10.4049/jimmunol.171.2.979

[30] Takahashi K, Yamaguchi S, Shimoyama T, Seki H, Miyokawa K, et al (2008) JNK-and IκB-dependent pathways regulate MCP1 but not adiponectin release from artificially hypertrophied 3T3-L1 adipocytes preloaded with palmitate in vitro. Am J Physiol Endocrinol Metab 294: E898–E909. 18303122 10.1152/ajpendo.00131.2007

[31] Hsueh HW, Zhou Z, Whelan J, Allen KG, Moustaid-Moussa N, et al (2011) Stearidonic and eicosapentaenoic acids inhibit interleukin-6 expression in ob/ob mouse adipose stem cells via toll-like receptor-2–Mediated pathways. J Nutr 141: 1260–1266. 21562237 10.3945/jn.110.132571

[32] Mosser DM, Edwards JP (2008) Exploring the full spectrum of macrophage activation. Nat Rev Immunol 8: 958–969. 19029990 10.1038/nri2448 PMC2724991

[33] Pascual G, Fong AL, Ogawa S, Gamliel A, Li AC, et al (2005) A SUMOylation-dependent pathway mediates transrepression of inflammatory response genes by PPAR-γ. Nature 437: 759–763. 16127449 10.1038/nature03988 PMC1464798

[34] Greene M, Blumberg B, McBride O, Yi H, Kronquist K, et al (1995) Isolation of the human peroxisome proliferator activated receptor gamma cDNA: Expression in hematopoietic cells and chromosomal mapping. Gene Expr 4: 281–289. 7787419 PMC6134382

[35] Martin H (2010) Role of PPAR-gamma in inflammation. Prospects for therapeutic intervention by food components. Mutat Res. 690: 57–63. 20973164 10.1016/j.mrfmmm.2009.09.009

[36] Kadegowda A, Bionaz M, Piperova L, Erdman R, Loor J (2009) Peroxisome proliferator-activated receptor-γ activation and long-chain fatty acids alter lipogenic gene networks in bovine mammary epithelial cells to various extents. J Dairy Sci 92: 4276–4289. 19700688 10.3168/jds.2008-1932

[37] Lolmède K, Duffaut C, Zakaroff-Girard A, Bouloumié A (2011) Immune cells in adipose tissue: Key players in metabolic disorders. Diabetes Metab 37: 283–290. 21507694 10.1016/j.diabet.2011.03.002

[38] Titos E, Rius B, González-Périz A, López-Vicario C, Morán-Salvador E, et al (2011) Resolvin D1 and its precursor docosahexaenoic acid promote resolution of adipose tissue inflammation by eliciting macrophage polarization toward an M2-like phenotype. JImmunol 187: 5408–5418. 22013115 10.4049/jimmunol.1100225

[39] Welch JS, Ricote M, Akiyama TE, Gonzalez FJ, Glass CK (2003) PPARγ and PPARδ negatively regulate specific subsets of lipopolysaccharide and IFN-γ target genes in macrophages. Proc Natl Acad Sci USA 100: 6712–6717. 12740443 10.1073/pnas.1031789100 PMC164512

[40] Odegaard JI, Ricardo-Gonzalez RR, Goforth MH, Morel CR, Subramanian V, et al (2007) Macrophage-specific PPARgamma; controls alternative activation and improves insulin resistance. Nature 447: 1116–1120. 17515919 10.1038/nature05894 PMC2587297

[41] Thieringer R, Fenyk-Melody JE, Le Grand CB, Shelton BA, Detmers PA, et al (2000) Activation of peroxisome proliferator-activated receptor γ does not inhibit IL-6 or TNF-α responses of macrophages to lipopolysaccharide in vitro or in vivo. J Immunol 164: 1046–1054. 10623855 10.4049/jimmunol.164.2.1046

[42] Choi JH, Banks AS, Kamenecka TM, Busby SA, Chalmers MJ, et al (2011) Antidiabetic actions of a non-agonist PPARγ ligand blocking Cdk5-mediated phosphorylation. Nature 477: 477–481. 21892191 10.1038/nature10383 PMC3179551

[43] Varga T, Czimmerer Z, Nagy L (2011) PPARs are a unique set of fatty acid regulated transcription factors controlling both lipid metabolism and inflammation. Biochim Biophys Acta 1812: 1007–1022. 21382489 10.1016/j.bbadis.2011.02.014 PMC3117990

[44] Lee JY, Sohn KH, Rhee SH, Hwang D (2001) Saturated fatty acids, but not unsaturated fatty acids, induce the expression of cyclooxygenase-2 mediated through toll-like receptor 4. J Biol Chem 276: 16683–16689. 11278967 10.1074/jbc.M011695200

[45] Han CY, Kargi AY, Omer M, Chan CK, Wabitsch M, et al (2010) Differential effect of saturated and unsaturated free fatty acids on the generation of monocyte adhesion and chemotactic factors by adipocytes dissociation of adipocyte hypertrophy from inflammation. Diabetes 59: 386–396. 19934003 10.2337/db09-0925 PMC2809975

[46] Bradley RL, Fisher FM, Maratos-Flier E (2008) Dietary fatty acids differentially regulate production of TNF-α and IL-10 by murine 3T3-L1 adipocytes. Obesity 16: 938–944. 18356844 10.1038/oby.2008.39 PMC4862864

