# Supplementary figures and images for: Docosahexaenoic Acid Decreases Pro-Inflammatory Mediators in an In Vitro Murine Adipocyte Macrophage Co-Culture Model
Source: PLoS One. 2014 Jan 20;9(1):e85037. doi: 10.1371/journal.pone.0085037 (PMC3896343; doi:10.1371/journal.pone.0085037)

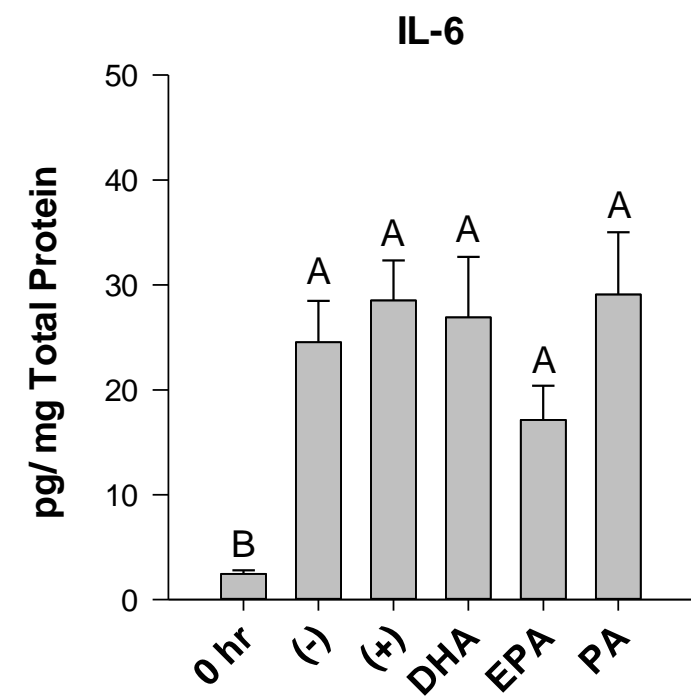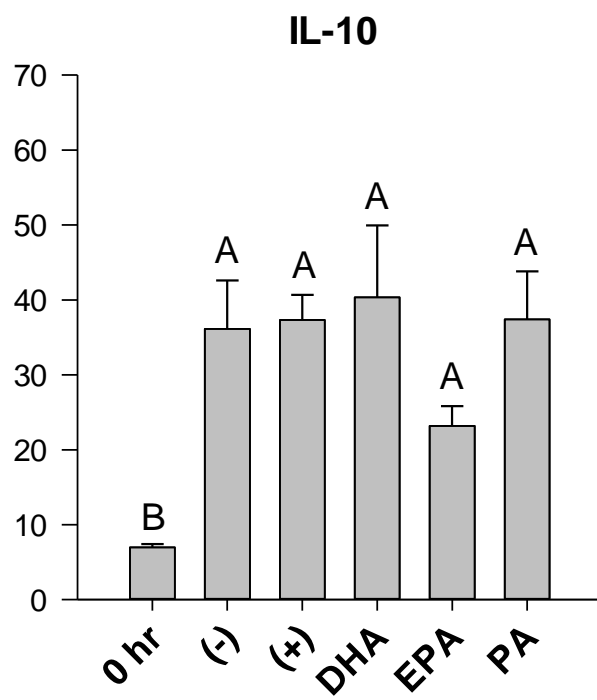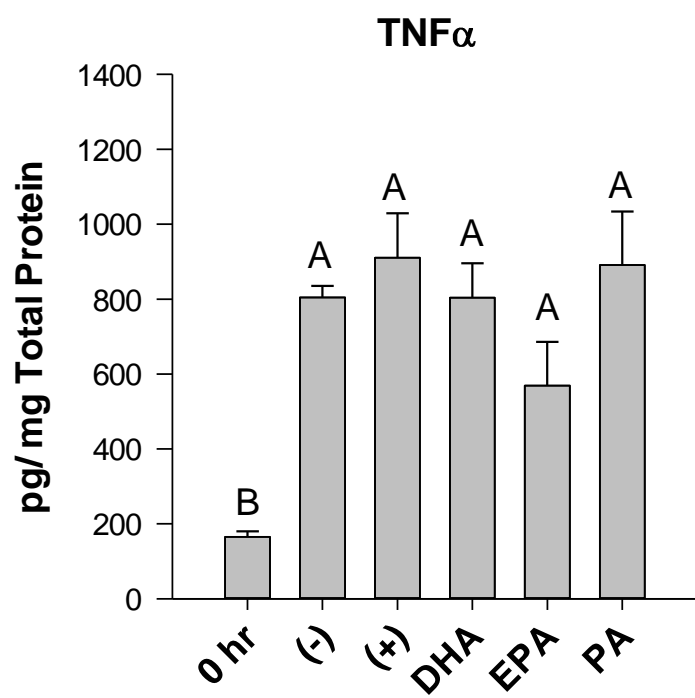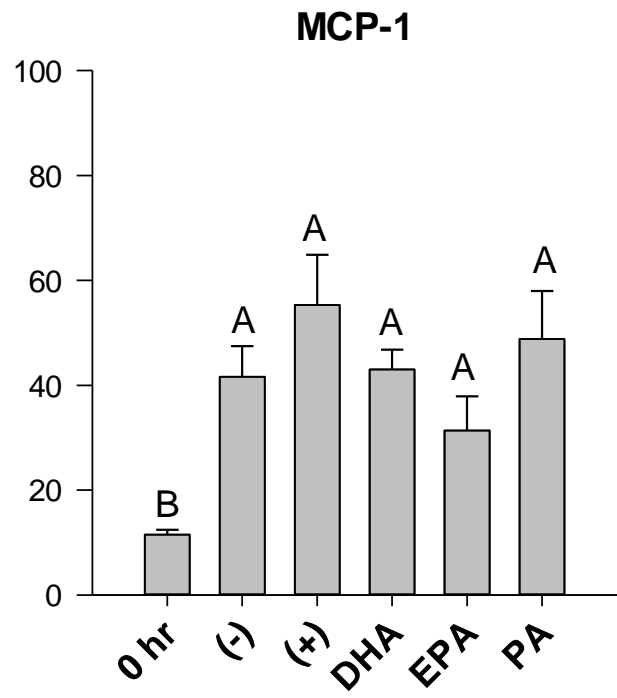

Supplement: Figure S1 — Adipocyte cellular protein in the trans-well system. The cellular protein concentrations of key cytokines (IL-6, MCP-1, TNFα and IL-10) measured from adipocytes in the trans-well system at 12 hr. 0 hr = serum starved adipocytes alone prior to co-culture and fatty acid treatment, (−) = negative control; adipocytes alone treated with 25 µM BSA, (+) = positive control; co-cultured adipocytes and macrophages plus 25 µM BSA, DHA = co-cultured adipocytes and macrophages in the presence of 125 µM DHA, EPA = co-cultured adipocytes and macrophages in the presence of 125 µM EPA, and PA = co-cultured adipocytes and macrophages in the presence of 125 µM PA. Values are means ± SEM. The experiment was independently conducted 2 times (in triplicate) for a final sample size of n = 6. A different letter indicates treatments are significantly different from each other, p≤0.05. (PDF) [file pone.0085037.s001.pdf]
